# Sleep disturbance and problematic alcohol use: Examination of sex and race differences

**DOI:** 10.3389/frsle.2022.1014610

**Published:** 2022-11-16

**Authors:** Joseph M. Dzierzewski, Scott G. Ravyts, Caitlin E. Martin, Kathryn M. Polak, Spencer A. Nielson, David Pomm, Pamela Dillon, Thomas B. Moore, Leroy R. Thacker, Dace S. Svikis

**Affiliations:** ^1^National Sleep Foundation, Washington, DC, United States; ^2^Department of Psychology, Virginia Commonwealth University, Richmond, VA, United States; ^3^Department of Obstetrics and Gynecology, School of Medicine, Virginia Commonwealth University, Richmond, VA, United States; ^4^Institute for Drug and Alcohol Studies, Virginia Commonwealth University, Richmond, VA, United States; ^5^Wright Center for Clinical and Translational Research, Virginia Commonwealth University, Richmond, VA, United States; ^6^Department of Biostatistics, School of Medicine, Virginia Commonwealth University, Richmond, VA, United States

**Keywords:** sleep, sleep disturbance, alcohol use, problematic drinking, sex differences, race differences

## Abstract

**Objectives:**

Disrupted sleep is prevalent and related to problematic alcohol use. While sex and race disparities exist in both sleep disturbances and problematic alcohol use, whether the association between disrupted sleep and problematic alcohol use is similar across sex and race is unknown. The present study sought to examine sex and race invariance in the association between disrupted sleep and problematic alcohol use.

**Methods:**

Secondary analyses of baseline data from a randomized clinical trial targeting heavy drinking in primary care facilities. Participants completed four individual sleep questions (sleep quality, difficulty falling asleep, difficulty staying asleep, and sleep medication use), along with sex-specific measures of problematic alcohol use (i.e., CAGE and T-ACE). A structural equation model (SEM) was used to examine the association between a latent sleep disturbance construct and problematic alcohol use, as well as potential sex and race invariance of this association.

**Results:**

Participants included 1,448 adults (76.59% female, 76.73% Black, *M*_age_ = 44.78, *SD* = 12.35). The majority of the sample reported one or more sleep disturbance symptoms and 31.84% of participants screened positive for problematic alcohol use. Greater sleep disturbance was significantly associated with a greater risk of problematic alcohol use (β = 0.18, *p* < 0.001), and did not differ by either sex or race.

**Conclusions:**

Disrupted sleep is associated with problematic alcohol use, across sex and race. Sleep interventions may hold promise as treatment augments in individuals with problematic alcohol use.

## Introduction

Disturbed sleep is very common, with 30% of the US population endorsing one or more symptoms of insomnia (Roth, 2007). Problematic alcohol use is also very prevalent, with estimates ranging between 7 and 20% of the population reporting drinking behaviors considered to be problematic or “risky” by clinical standards (Saitz, 2005). Primary care providers are responsible for the bulk of care related to both insomnia (Shochat et al., 1999) and problematic alcohol use (Buchsbaum et al., 1991). While there are known sex and race disparities in both insomnia rates (Zhang and Wing, 2006; Kaufmann et al., 2016) and problematic alcohol use (Barr et al., 1993; Nolen-Hoeksema and Hilt, 2006), whether disparities exist in the association between insomnia symptoms and problematic alcohol use is unknown and inconsistently reported in the literature (Inkelis et al., 2020). The current investigation aimed to investigate the association between insomnia symptoms and problematic alcohol use, along with investigating both sex and race differences in this important association among a large, diverse sample of individuals presenting to a routine primary care visit.

Disturbed sleep, as indicated by the presence of insomnia symptoms [i.e., difficulty falling asleep, difficulty staying asleep, or experiencing poor sleep quality (i.e., non-restorative sleep)], is very prevalent with estimates suggesting that 30% of the population reports one or more symptoms of insomnia (Roth, 2007). While the rates of disturbed sleep are high in the general US populations, higher rates of insomnia symptoms have been noted in women (Zhang and Wing, 2006), racial and ethnic minorities (Kaufmann et al., 2016), older adults (Dzierzewski et al., 2018), and those with mental and physical health conditions (Kay and Dzierzewski, 2015). Unsurprisingly, disturbed sleep is also more prevalent among individuals presenting for care at primary care offices (Shochat et al., 1999).

In terms of problematic alcohol use, a very similar pattern emerges as that previously described for disturbed sleep. In the general population, problematic alcohol use, as defined by the presence of any two of the following: annoyance over people criticizing drinking, guilt over drinking, consumption of an eye opener drink, and either a desire to cut down or tolerance, has estimated prevalence rates between 7 and 20% (Saitz, 2005). When examining rates of problematic alcohol use in subsegments of the population, there are notable disparities by sex (Nolen-Hoeksema and Hilt, 2006), race (Barr et al., 1993), age (Livingston and Room, 2009), and socioeconomic status (Calling et al., 2019), among others. Similar to disrupted sleep, higher rates of problematic alcohol use are observed in primary care facilities (Gupman et al., 2002; Rehm et al., 2016).

Disturbed sleep and problematic alcohol use have a reciprocal relationship (Haario et al., 2013; Fucito et al., 2018; Rognmo et al., 2019; Inkelis et al., 2020), with insomnia symptoms being associated with increased risk for problematic alcohol use (Roehrs and Roth, 2018) and recurrence of use among individuals with alcohol use disorder (Chakravorty et al., 2016), as well as problematic alcohol use being a risk for disturbed sleep (Colrain et al., 2009). As summarized above, there are known disparities in rates of both disturbed sleep and problematic alcohol use; however, less is known regarding potential disparities in the associations between insomnia symptoms and problematic alcohol use (Hu et al., 2020; Inkelis et al., 2020). Previous research examining sleep and alcohol use has typically not had the needed sample size or sample characteristics required to examine sex or race differences in this important association. Studies that have directly addressed sex disparities in the alcohol-sleep association have reported inconsistent findings, with some reporting significant negative associations between alcohol and sleep only in men (Rognmo et al., 2019), significant negative associations between alcohol and sleep only in women (Verlinden et al., 2022), or significant positive associations between alcohol and sleep only in women (Freeman et al., 2022). In fact, the majority of what is known regarding the insomnia symptoms—problematic alcohol use association is drawn from comparisons across studies (Inkelis et al., 2020), a practice that can distort conclusions due to sample-, context-, and study-specific characteristics.

The current study aimed to address these shortcomings through investigating the link between insomnia symptoms and problematic alcohol use in a large, sex and race diverse, sample of adults. First, we aimed to examine the association between insomnia symptoms and problematic alcohol use in a sample of adults presenting to primary care facilities for routine care. Next, we aimed to determine whether the association between insomnia symptoms and problematic alcohol is invariant across sex (men and women) and race (Black and White). We hypothesized that individuals with worse insomnia symptoms would have greater risk of problematic alcohol use and that this association would be invariant across sex and race.

## Materials and methods

### Design and procedures

These analyses are secondary analyses of baseline data from a randomized clinical trial targeting heavy drinking in primary care facilities. Patients were recruited from urban primary care and gynecologic clinics within a university-based health system. Patients were recruited in clinic waiting areas for an anonymous survey focused on health behaviors. Interested participants were escorted to a private area adjacent to the clinic waiting room either before or after their scheduled medical appointments. To participate in the survey, inclusion criteria were limited to age ≥18 years and ability to understand spoken and written English. After providing verbal informed consent, participants completed the 15-mins Health Cheq survey on a tablet PC and wearing headphones. An intervention authoring tool, developed for previous work, was used to design and deliver the survey. Participants were guided through the survey by a 3-D avatar (Peedy the Parrot) who read each question aloud and kept participants engaged in the process (Ondersma et al., 2005; Breland et al., 2014). Patients received $10 for their participation in the study which was reviewed and approved by the Virginia Commonwealth University Institutional Review Board.

### Measures

Health Cheq collected information on patient demographics, general health behaviors, general medical concerns, mental health, and psychosocial issues (Kelpin et al., 2018). The current study analyzed data from the following survey domains:

*Demographics*. Demographic variables included sex, age, race, and education.

*Problematic alcohol use*. Health Cheq collected the standard Cut down, Annoyed, Guilty, and Eye-opener (CAGE) questionnaire for alcohol use if male (Ewing, 1984) or the Tolerance, Annoyance, Cut down, and Eye-opener (T-ACE) questionnaire if female (Stevenson and Masters, 2005). The T-ACE was adapted from the CAGE when it was observed that the CAGE was potentially limited in its sensitivity in detecting alcohol use in women (Stevenson and Masters, 2005). These measures are largely similar and only differ by one question. A positive screen for both measures is defined as a total score of two or greater (i.e., at least two items endorsed on the CAGE or T-ACE, or the single tolerance item endorsed on the T-ACE).

*Sleep disturbance*. To assess sleep quality, participants were asked: “How would you rate the quality of your sleep in the past 30 days?” (with response options: Very Good, Good, Fair, and Poor) based on an item from the Pittsburgh Sleep Quality Index (PSQI) (Buysse et al., 1989). Difficulty falling asleep (“In the past 30 days, did you have trouble falling asleep?”) and difficulty staying asleep (“In the past 30 days, did you have trouble staying asleep?”) were assessed using items derived from the Insomnia Severity Index (Bastien et al., 2001), but were assessed with a dichotomous answer choice (i.e., “yes” or “no”). Sleep medication use was assessed with the following item based on a question from the PSQI (Buysse et al., 1989): “In the past 30 days, did you take anything to help you sleep?” (with response options: No; Yes, I take prescription medications; Yes, I take non-prescription medications; and Yes, I take both prescription and non-prescription medications).

### Data analysis

Data were analyzed using SPSS v.27 and AMOS v.27 (Arbuckle, 2020; IBM SPSS Statistics for Windows, 2020). In order to assess the association between sleep and alcohol use, a series of binary logistic regressions were run to estimate the odds ratio (ORs) and 95% confidence intervals (CIs) of problematic alcohol use (operationalized as a positive screen on the CAGE or T-ACE). A structural equation model (SEM) was also used to examine the association between sleep disturbance and problematic alcohol use. Sleep disturbance was conceptualized as a latent construct consisting of items related to sleep quality, difficulty falling asleep, difficulty staying asleep, and sleep medication use (including prescription and non-prescription use), with higher scores indicating worse sleep. Problematic alcohol use was defined as a positive screen on either the CAGE or T-ACE (depending on a participant's sex). Goodness of fit for the SEM was assessed using well established guidelines (Bentler, 1990; Byrne, 1994; Tabachnick et al., 2007). A complete CAGE or T-ACE was needed to be included in the present analysis; other missing data was handled using full informal maximum likelihood (FIML) (Enders and Bandalos, 2001). Finally, two invariance analyses examined whether the association between sleep disturbance and problematic alcohol use varied as a function of sex (male vs. female) or race (White vs. Black). *Post-hoc* comparisons of non-invariance were completed using Bonferroni-corrections which accounted for the number of variables correlating with the latent sleep disturbance construct.

## Results

### Participants descriptive statistics

The total sample consisted of 1,448 participants. Participants were predominately female (76.59%), Black (76.73%), and middle-aged (*M* = 44.78, *SD* = 12.35). The most common level of education for participants was a high school degree or GED (26.86%). Sleep was poor with over half of participants endorsing difficulty falling sleep (59.46%), nearly two-thirds reporting difficulty staying asleep (64.02%), and slightly less than one third of the sample endorsing poor to fair sleep quality (32.80%). Over a quarter of the sample (25.45%) reported taking prescription sleep medication in the past 30 days, while 13.91% reported taking non-prescription sleep medication. Basic descriptive and clinical statistics are presented in Table 1.

**Table 1:** Participants descriptive statistics (*N* = 1,448).

	***N* (*%*)**
Age, mean (*SD*)	44.78 (12.35)
Sex	
Male	339 (23.41%)
Female	1,109 (76.59%)
Race	
White	337 (23.27%)
Black	1,111 (76.73%)
Sleep quality	
Very good	121 (8.36%)
Good	407 (28.11%)
Fair	541 (37.36%)
Poor	379 (26.17%)
Trouble falling asleep	861 (59.46%)
Trouble staying asleep	927 (64.02%)
Sleep medication use	
None	1,006 (64.49%)
Prescription	397 (25.45%)
Non-prescription	217 (13.91%)
CAGE, Mean (*SD*)	1.36 (1.41)
T-ACE, Mean (*SD*)	1.18 (1.09)
General health	
Excellent	63 (6.63%)
Very good	331 (22.86%)
Good	546 (37.71%)
Fair	389 (26.86%)
Poor	86 (5.94%)

Age measured in years. Trouble falling asleep and trouble staying asleep were assessed using a yes/no response.

None of the sleep variables differed by sex (*p'*s > 0.05). By contrast, while White and Black participants did not have statistically different levels of sleep quality (*p* = 0.08) or difficulty falling asleep (*p* = 0.50), White participants were more likely than Black participants to take sleep medications (44.81 vs. 33.39%; χ^2^ (1, *N* = 1,448) = 15.60, *p* < 0.001) and endorse difficulty staying asleep (68.54 vs. 62.65%; χ^2^ (1, *N* = 1,448) = 3.91, *p* = 0.048).

Overall, 31.84% of participants screened positive for problematic alcohol use. Males were significantly more likely than women to screen positive for problematic alcohol use, χ^2^ (1, *N* = 1448) = 7.88, *p* = 0.01, with 38.05 and 29.94% of males and females screening positive on the CAGE and T-ACE, respectively. Problematic alcohol use was also significantly higher among Black (33.39%) than White participants (26.71%), χ^2^ (1, *N* = 1448) = 5.33, *p* = 0.02. Sex and racial differences in sleep and problematic alcohol use are presented in Table 2.

**Table 2:** Sex and racial differences in sleep and problematic alcohol use.

	**Male**	**Female**	***p* value**
Sleep quality, Mean (SD)	1.84 (0.93)	1.18 (0.92)	0.53
Trouble falling asleep	195 (57.52%)	666 (60.05%)	0.41
Trouble staying asleep	216 (63.72%)	711 (61.93%)	0.89
Sleep medication use	115 (33.92%)	403 (36.33%)	0.42
Problematic alcohol use	129 (38.05%)	332 (29.94%)	0.01
	**White**	**Black**	***p*** **value**
Sleep quality, mean (SD)	1.74 (0.96)	1.84 (0.90)	0.08
Trouble falling asleep	195 (57.86%)	666 (59.95%)	0.50
Trouble staying asleep	231 (68.54%)	696 (62.65%)	0.048
Sleep medication use	151 (44.81%)	367 (33.03%)	<0.001
Problematic alcohol use	90 (26.71%)	371 (33.39%)	0.02

Problematic alcohol use is operationalized as positive screen on either the CAGE or T-ACE depending on sex.

Participants who endorsed either difficulty falling asleep (OR = 1.82, 95% CI = 1.44, 2.30), difficulty staying asleep (OR = 1.70, 95% CI = 1.37, 2.17), or prescription sleep medication use (OR = 1.72, 95% CI = 1.37, 2.17) were more likely to screen positive for problematic alcohol use. Similarly, individuals who screened positive for problematic alcohol use were more likely to report poorer sleep quality, [*F*_(1,1446)_ = 32.63], *p* < 0.001.

### Model fit

The chi-squared test was non-significant, χ^2^ (5) = 8.27, *p* = 0.14, indicating good global model fit. Moreover, the incremental fit index (IFI), Tucker-Lewis index (TLI), and comparative fit index (CFI) were all above 0.98 where values above 0.95 indicate good fit. The current model also produced a root mean square error of approximation (RMSEA) of 0.02, where an RMSEA of 0.08 or less indicates a reasonable error of approximation and adequate fit. Taken together, these goodness-of-fit indices suggested that the model adequately fit the data.

### Structural model

**Figure 1:**
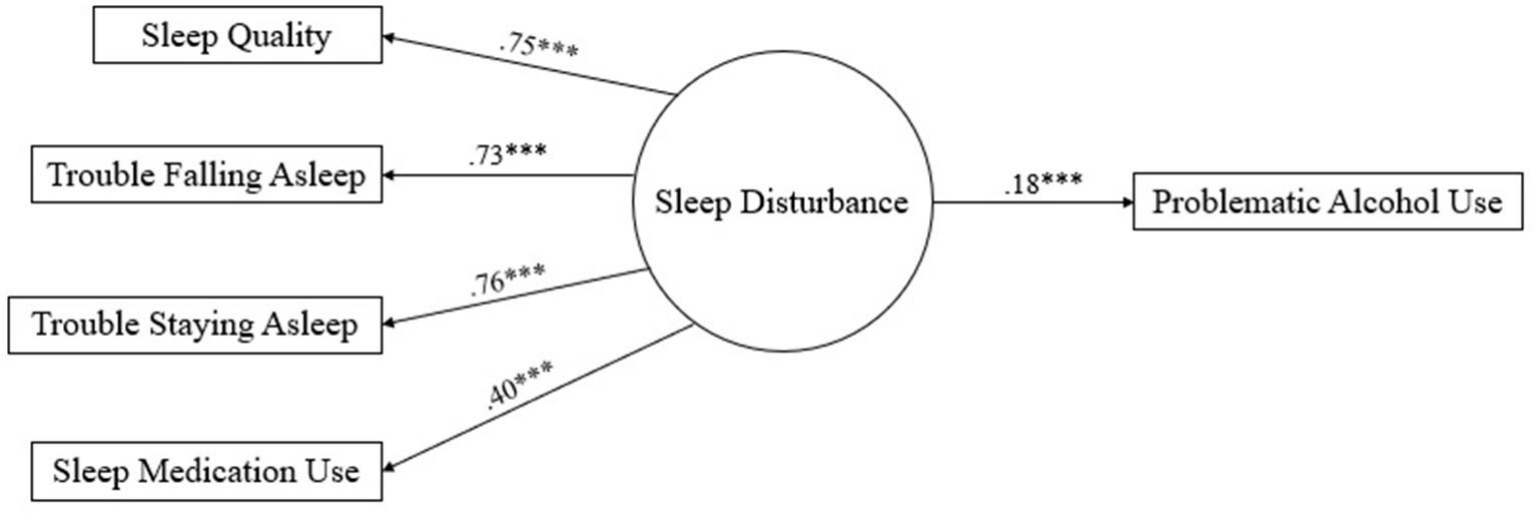
Structural equation model for sleep disturbance predicting problematic alcohol use. Latent variables are represented by circles and manifest variables are presented by rectangles; values next to each arrow represents the value of the standardized regression weights; higher scores indicate worse sleep quality *** *p* < 0.001.

The structural model revealed that sleep quality, trouble falling asleep, trouble staying asleep, and sleep medication use (including prescription and non-prescription use) all loaded significantly onto the latent variable of sleep disturbance (*ps* < 0.001), with higher scores indicating worse overall sleep. Additionally, greater sleep disturbance was significantly associated with a greater risk of problematic alcohol use (β = 0.18, *p* < 0.001) accounting for 3% of the variance. The complete SEM is presented in Figure 1.

### Invariance analyses

Invariance analyses for sex (male vs. female) and race (White vs. Black) evaluated the difference between an unconstrained model, which assumes that the groups yield different values of the parameters when the model is applied to the data, to a structural weights model, which assumes that the groups yield equivalent path coefficients when the model is applied to the data. For sex analyses, the unconstrained model yielded a non-statistically significant chi-square value *X*^2^ (10, *N* = 1,448) = 17.92, (*p* = 0.06). The chi-square difference test for the measurement weights model was similarly non-significant, *X*^2^ (4, *N* = 1,448) = 3.11, (*p* = 0.54). Thus, these results suggest invariance across sex with regards to the pattern coefficients associating indictor variables to factors.

For invariance analyses pertaining to race, the unconstrained model yielded a non-statistically significant chi-square value *X*^2^ (10, *N* = 1,448) = 10.22, (*p* = 0.42). Contrasted to the unconstrained model, the chi-square difference test for the measurement weights model was significant, *X*^2^ (4, *N* = 1,448) = 14.60, (*p* = 0.002). These results suggest non-invariance across race with regards to the pattern coefficients associating the indictor variables to their factor. Bonferroni-corrected *post-hoc* analyses, accounting for the number of variables correlating with the latent construct of sleep disturbance in the model, revealed a significant difference in the factor loading of sleep quality. Specifically, sleep quality loaded more strongly onto sleep disturbance for White (*B* = 0.81) than Black (*B* = 0.74) participants (z = −2.56, *p* = 0.01). Importantly, the association between sleep disturbance and problematic alcohol use did not differ by race (*z* = −0.37, *p* > 0.05).

## Discussion

In this sample of adults seeking care within a primary care setting, both sleep disturbance symptoms and problematic alcohol use were common. Further, self-reported sleep disturbance symptoms were associated with a positive screen for problematic alcohol use. Despite disparities in both sleep health and substance use documented by sex and race in the general population, this association between sleep disturbance and alcohol remained consistent across these groups within this primary care sample. Overall, study findings indicate the need for further exploration of how substance use and sleep, two critical areas of health, intersect in order to inform culturally tailored advancements in clinical care and public health strategies.

Alcohol has well known negative effects on sleep across multiple parameters, but the intersection of sleep with problematic alcohol use and alcohol use disorder (AUD) remains less understood. The mechanism by which unhealthy sleep leads to problematic alcohol use or vice versa is likely complex and varies across an individual's biopsychosocial factors. The neural mechanisms involved in sleep overlap substantially with those in the reward system (Valentino and Volkow, 2020). For individuals at risk, these crossing neurobiological pathways can lead to a vicious cycle where substance use and sleep disturbance continue to perpetuate one another (Koob et al., 2020). For example, poor sleep can lead to increased impulsivity and decreased attention, risk factors for persistent alcohol use despite adverse consequences (Roehrs et al., 2021). Simultaneously, as the reward system becomes increasingly impaired in the setting of chronic alcohol use, a hypernegative emotional state predominates, characterized by sleep disturbances and other symptoms such as dysphoria (Koob, 2021). Our data elucidating significant associations between sleep disturbance and problematic alcohol use using validated screening tools likely are a snapshot of this ongoing cycle at a single time point. Nonetheless, sleep and alcohol use are intertwined, and the chronology of their impairments should be further elucidated using longitudinal data in future research.

In the primary care setting, insomnia is actively being targeted for innovative, large scale interventions given existing robust evidence of its impacts on health across multiple domains (Sweetman et al., 2021). Study findings indicating a persistent interplay between sleep and problematic alcohol use across clinical subgroups highlight the importance of integrating alcohol use assessments and interventions into sleep-focused implementation trials. Also, given how both alcohol use itself and the impaired neurocognitive functions in the setting of AUD negatively impact sleep, findings also indicate that these emerging insomnia treatment pathways would likely achieve better long-term patient outcomes by co-addressing problematic alcohol use with insomnia. For example, coupling sleep and alcohol assessments and treatments, such as pathways to integrate cognitive behavioral therapy for insomnia with evidence-based SBIRT (Screening, Brief Intervention, Referral to Treatment) (Babor et al., 2017) approaches for alcohol use could be an effective means to achieve optimal outcomes for both conditions. Prior evidence in primary care settings demonstrating how addressing sleep can facilitate also addressing substance use (Fortuna et al., 2018), a highly stigmatized topic, also support such future studies.

Similarly, study findings also indicate the potential sleep has as a target for improved patient outcomes across racial groups and sex in the setting of addressing problematic alcohol use and AUD. Poor sleep quality, trouble falling asleep, trouble staying asleep and sleep medication use all demonstrated significant associations with problematic alcohol use in our sample of predominantly Black female primary care patients. Prior literature among individuals with AUD do not indicate a clear pattern of sleep indices associated with continued or recurrence of heavy alcohol use (Kolla et al., 2015). However, insomnia symptoms have been associated with increased cravings and increased risk of relapse (Brower et al., 1998; Conroy et al., 2006; He et al., 2019), dysfunctional sleep behaviors or thoughts increase the risk for alcohol use recurrence (Brooks et al., 2021), and a history of insomnia predicts alcohol use following substance use disorder treatment (Dolsen and Harvey, 2017). AUD has devastating health consequences, and alcohol-related liver disease is a leading cause of death, with rapidly increasing mortality rates among females over the past decade (Woolf and Schoomaker, 2019). However, only about 1 in 7 individuals with AUD receive treatment in the United States, with <2% receiving evidence-based AUD medications (Han et al., 2021). Thus, in addition to an urgent need to improve AUD treatment expansion and engagement, optimizing AUD treatment retention and outcomes are imperative. Integrating patient centered interventions focused on sleep health into AUD treatment settings could be an effective means to meet these needs. For example, cognitive behavioral therapy for insomnia (CBT-I), the frontline evidence-based approach to treating insomnia, may be a fruitful treatment approach to integrate within current treatment paradigms for AUD treatment-seeking individuals (Brooks et al., 2018; Miller et al., 2021). Indeed, three clinical trials investigating the effect of cognitive behavioral therapy for insomnia (CBT-I) for individuals with alcohol dependency and those who were recovering from alcohol dependency have been conducted (Currie et al., 2004; Arnedt et al., 2011; Chakravorty et al., 2019). These trials observed that CBT-I was effective in reducing insomnia symptoms and improving daytime functioning in these samples but also observed little to no effects on drinking behaviors. However, these trials were not sufficiently powered to fully investigate the effects of CBT-I on drinking behaviors (Arnedt et al., 2022). As such, more research is needed to inform the development of pharmacological and non-pharmacological options to treat insomnia that are tailored to the unique needs of adults with AUD (Miller et al., 2017; Roehrs et al., 2020).

This study has several potential limitations and strengths. First, the measurement of sleep was limited to four self-report questions about insomnia symptoms, and a validated measure of sleep disturbance, or measures of other sleep difficulties such as sleep-disordered breathing, were not used. However, the questions used in this study measured key symptoms of sleep disturbance (i.e., sleep quality, difficulty falling and staying asleep, and use of medication) demonstrating high face validity. Further, the questions were shown to reflect a latent construct of sleep disturbance, thereby demonstrating that they were most likely measuring sleep disturbance. Further research using validated measures of sleep disturbance, insomnia, and other sleep difficulties, such as sleep-disordered breathing, may help elucidate underlying associations. Another potential limitation of the current study is that problematic alcohol use was measured through retrospective self-report, which may introduce bias. Moreover, these measures utilized a dichotomous yes/no question format which can be less informative than a continuous measure of alcohol use. Future studies may benefit from using a continuous measure of alcohol use or a prospective daily diary approach, measuring daily sleep and alcohol use to examine daily associations between sleep disturbance and alcohol use. Lastly, due to the cross-sectional nature of this study, we were unable to examine directionality between sleep disturbance and alcohol use. Key strengths of the study include the large sample, high proportion of women and Black participants, and use of sex-specific measures of problematic alcohol use.

In conclusion, this study examined associations between self-reported sleep disturbance symptoms and problematic alcohol use in a large primary care sample that was both sex and race diverse. Sleep disturbance and problematic alcohol use were significantly associated, and this association was consistent across sex and race. The association between sleep disturbance and problematic alcohol use and the high prevalence of sleep disturbances within problematic alcohol use identifies sleep disturbance as a potential treatment target to consider when caring for individuals with AUD. Further research into the intersection between sleep disturbance and alcohol use is needed to elucidate whether targeting sleep disturbance in AUD treatment may help achieve better long-term patient outcomes.

## Data availability statement

The raw data supporting the conclusions of this article will be made available by reasonable request to the corresponding author.

## Ethics statement

The studies involving human participants were reviewed and approved by Institutional Review Board, Virginia Commonwealth University. The patients/participants provided their verbal informed consent to participate in this study.

## Author contributions

JD, SR, CM, KP, and SN: drafted portions of the manuscript and drafted a critical review. SR and JD: analysis. DP, PD, TM, LT, and DS: critical review. KP, DP, TM, and DS: data collection. PD, LT, and DS: conceptualization, design, and funding. All authors contributed to the article and approved the submitted version.

## Funding

Research reported in this publication was supported by the National Institute on Aging under Grant (K23AG049955, PI: JD), National Institute on Drug Abuse under Grant (K23DA053507, PI: CM), National Institute on Drug and Alcohol Abuse under Grant (R01DA026091, PI: DS), and CTSA award from the Center for Advancing Translational Sciences (UL1TR002649, PI: Moeller).

## Conflict of interest

The authors declare that the research was conducted in the absence of any commercial or financial relationships that could be construed as a potential conflict of interest.

## Publisher's note

All claims expressed in this article are solely those of the authors and do not necessarily represent those of their affiliated organizations, or those of the publisher, the editors and the reviewers. Any product that may be evaluated in this article, or claim that may be made by its manufacturer, is not guaranteed or endorsed by the publisher.
